# Progestogen-induced alterations and their ecological relevance in different embryonic and adult behaviours of an invertebrate model species, the great pond snail (*Lymnaea stagnalis*)

**DOI:** 10.1007/s11356-020-12094-z

**Published:** 2020-12-21

**Authors:** Reka Svigruha, Istvan Fodor, Judit Padisak, Zsolt Pirger

**Affiliations:** 1 Department of Limnology, University of Pannonia, Veszprém, 8200 Hungary; 2 NAP Adaptive Neuroethology Research Group, Department of Experimental Zoology, Balaton Limnological Institute, Centre for Ecological Research, Tihany, 8237 Hungary

**Keywords:** Progestogen exposure, *Lymnaea stagnalis*, Developmental changes, Heartbeat, Locomotion, Feeding activity

## Abstract

The presence of oral contraceptives (basically applying estrogens and/or progestogens) poses a challenge to animals living in aquatic ecosystems and reflects a rapidly growing concern worldwide. However, there is still a lack in knowledge about the behavioural effects induced by progestogens on the non-target species including molluscs. In the present study, environmental progestogen concentrations were summarised. Knowing this data, we exposed a well-established invertebrate model species, the great pond snail (*Lymnaea stagnalis*) to relevant equi-concentrations (1, 10, 100, and 500 ng L^−1^) of mixtures of four progestogens (progesterone, drospirenone, gestodene, levonorgestrel) for 21 days. Significant alterations were observed in the embryonic development time, heart rate, feeding, and gliding activities of the embryos as well as in the feeding and locomotion activity of the adult specimens. All of the mixtures accelerated the embryonic development time and the gliding activity. Furthermore, the 10, 100, and 500 ng L^−1^ mixtures increased the heart rate and feeding activity of the embryos. The 10, 100, and 500 ng L^−1^ mixtures affected the feeding activity as well as the 1, 10, and 100 ng L^−1^ mixtures influenced the locomotion of the adult specimens. The differences of these adult behaviours showed a biphasic response to the progestogen exposure; however, they changed approximately in the opposite way. In case of feeding activity, this dose-response phenomenon can be identified as a hormesis response. Based on the authors’ best knowledge, this is the first study to investigate the non-reproductive effects of progestogens occurring also in the environment on molluscan species. Our findings contribute to the global understanding of the effects of human progestogens, as these potential disruptors can influence the behavioural activities of non-target aquatic species. Future research should aim to understand the potential mechanisms (e.g., receptors, signal pathways) of progestogens induced behavioural alterations.

**Supplementary Information:**

The online version contains supplementary material available at 10.1007/s11356-020-12094-z.

## Introduction

In the last few years, it has become clear that pharmacologically active compounds (PhACs), as emerging pollutants in aquatic ecosystems, pose a challenge to animals and has initiated a rapidly growing concern on their environmental impact (Can et al. 2014; Liu et al. 2011a; Maasz et al. 2019; Postigo et al. 2010). One such group of PhACs are the synthetic oral contraceptives (basically applying estrogens and/or progestogens). For a long time, the estrogens were the most extensively studied contraceptive compounds; their effects have been shown on different invertebrate and vertebrate aquatic species (Bhandari et al. 2015; Caldwell et al. 2008; Costa et al. 2010; Huang et al. 2015; Hutchinson 2002; Islam et al. 2020; Kashian and Dodson 2004; Ketata et al. 2008; Matthiessen and Sumpter 1998; Torres et al. 2015; Zou and Fingerman 1997). Recently, another class of contraceptive pharmaceuticals has emerged into the focus in ecotoxicology: the progesterone (PRG) and its synthetic analogue progestins (e.g. drospirenone [DRO], gestodene [GES], and levonorgestrel [LNG]); generally referred to as progestogens (Sitruk-Ware and Nath 2010). In previous studies, PRO, DRO, GES, and LNG were detected at concentrations of typically a few ng L^−1^ in surface waters (Chang et al. 2011; Fent 2015; Liu et al. 2011a, b; Orlando and Ellestad 2014; Shen et al. 2018; Vulliet et al. 2008; Yost et al. 2014). In our pilot study area, by analysing freshwater samples from the catchment area of the largest shallow lake in Central Europe, Lake Balaton, varying progestogen concentrations of 0.6–50 ng L^−1^ were detected (Avar et al. 2016; Maasz et al. 2019). Despite these relatively low environmental concentrations, progestogens have extreme stability against oxidation or degradation in the environment due to the polycyclic sterane frame and ethynyl-group (LNG, GES). Therefore, the continuous and simultaneous presence of these chemicals might be enough to impose a possible effect on non-target aquatic biota (Frankel et al. 2016; Giusti et al. 2014; Maasz et al. 2017; Tillmann et al. 2001; Zrinyi et al. 2017). To note, the large majoritIn the last few yearsy of these studies applies only a single PhAC in the laboratory experiments; however, there is a lack of information about the adverse mixture effects of progestogens, especially at average environmental (~ 1–10 ng L^−1^) concentrations.

Molluscs, which is the second most diverse animal group, are generally considered as excellent (bio)indicators (e.g. sensitive, easy to collect, and ubiquitous) of ecosystem health and frequently used for environmental studies. One such species is the great pond snail (*Lymnaea stagnalis*) that was found to be a sensitive and suitable species in ecotoxicological context. Several toxicological values and endpoints are available for *L. stagnalis* such as quantified reproductive, growth parameters, behavioural patterns as well as cellular and molecular biomarkers and/or responses (Amorim et al. 2019). The relevance of *L. stagnalis* in ecotoxicological studies is also supported historically: it became the first recognised, aquatic, non-arthropod invertebrate model organism in environmental risk assessments (Amorim et al. 2019; Ducrot et al. 2014; Giusti et al. 2014; Fodor et al. 2020a; Pirger et al. 2018). The developed standard reproduction and neurotoxicity tests of human drugs were officially approved by the national coordinators of the Organization for Economic Cooperation and Development (OECD 2016).

In our previous study, by investigating the effect of progestogens on the reproduction of *L. stagnalis,* we observed that parental progestogen exposure could cause alterations in the egg production of adult specimens as well as molecular and cellular changes in the early phase of embryonic development (Zrinyi et al. 2017). Here, our aim was to extend these pilot observations in an overarching investigation by monitoring different embryonic and adult behavioural parameters and possible underlying mechanisms at the cellular level. Such additional information could allow integrating findings into a complete picture of the mode of action of progestogens and pave the way for understanding the ecotoxic effects in more detail. To do so, embryos and adult specimens were exposed to different equi-concentrations (1, 10, 100, 500 ng L^−1^) of mixtures of four progestogens (PRG, DRO, GES, and LNG). Significant alterations in embryonic development time, heartbeat, feeding, and gliding activities and in adult feeding and locomotion activities were observed. Also, significant changes were determined in stress markers such as protein deglycase DJ-1 and p38alpha mitogen-activated protein kinase of the central nervous system (CNS).

## Materials and methods

### Chemicals

HPLC grade PRG (CAS No.: 57-83-0), LNG (CAS No.: 797-63-7), GES (CAS No.: 60282-87-3), and DRO (CAS No.: 67392-87-4) were used for the treatments as progestogen agents (Sigma-Aldrich, Hungary). Stock solutions of them (1 mg mL^−1^) were prepared in acetone (ACS reagent, ≥ 99.5%; CAS No.: 67-64-1; VWR, Hungary). From these stock solutions, 1 μg mL^−1^ working solutions were prepared (solvent at ≤ 0.01%). This working was added into the artificial snail water of experimental plastic well plates (embryos) or glass tanks (adults) with appropriate aliquots to reach the desired nominal equi-concentrations of 1, 10, 100, and 500 ng L^−1^ (mixtures of progestogens).

### Experimental animals and progestogen exposure

Adult (3–4 months old) specimens of *L. stagnalis*, originating from our laboratory-bred stocks (Balaton Limnological Institute, Tihany, Hungary), were randomly selected for use in our experiments. Snails were kept in large (20 L) holding tanks containing oxygenated low-copper artificial snail water at a constant temperature of 20 °C (± 1.5 °C) and on light:dark regime of 12 h:12 h. Specimens were fed on lettuce *ad libitum* three times a week and on vegetable-based fish food (TETRA Werke Company, Germany) one time a week. All procedures on snails were performed according to the protocols approved by the Scientific Committee of Animal Experimentation of the Balaton Limnological Institute (VE-I-001/01890-10/2013). Efforts were made to minimise the number of animals used in the experiments.

Adult snails were food-deprived for 2 days before the behavioural experiments. The experiments consisted of control and treated groups (12 adult animals/group/tank; *n* = 60 total number of adult animals per replicates) and data were obtained from 3 independent treatment series. Adult animals in the treated groups were exposed to 1, 10, 100, and 500 ng L^−1^ of mixtures of progestogens in 2 L artificial snail water for 3 weeks, respectively. Animals in the control group were kept in 2 L artificial snail water originally containing the solvent (≤ 0.000001%). Any physiological changes induced by the solvent cannot be observed. Based on our previous study (Zrinyi et al. 2017), water was totally refreshed weekly, and progestogens were added to reach the desired nominal concentrations again. During the 21-day exposure, highly paying attention to the same amount in the groups, specimens were fed on lettuce three times a week.

Egg masses were collected from the large tank of laboratory populations within 6 h after egg laying (cleavage period). Following previous studies (Filla et al. 2009; Voronezhskaya et al. 1999), we used isolated living embryos to ensure the appropriate tracking of behaviours and more standardised and reproducible experiments. Individual embryos were separated randomly from freshly laid egg masses and placed into 6-well plates (BioLite 6 Well Multidish; #100184 Thermo Fischer Scientific) with the following arrangement: *n* = 10 embryos/group/well in 10 mL oxygenated low-copper artificial snail water or progestogen-containing solutions. The experiments consisted of control and treated groups and data were obtained from 3 independent treatment series. Embryos in the control experiments were kept in 10 mL artificial snail water originally containing the solvent (≤ 0.0001%). No effects of the solvent were observed. Embryos in the treated groups were exposed to 1, 10, 100, and 500 ng L^−1^ of mixtures of PRG, LNG, GES, and DRO from cleavage period (E0 stage) to embryo hatching (E100 stage). Water and treated solutions were refreshed every 3 days.

### Observation of embryonic development

The development of *L. stagnalis* embryos generally takes place approx. 11–12 days in transparent eggs packaged in a translucent gelatinous mass, hence can be conveniently monitored by a stereomicroscope. The embryonic development was staged on the basis of a specific set of morphological, morphometric, and behavioural features, according to Morrill (Morrill 1982 ) and Mescheriakov (Mescheriakov 1990). Schematic (basic) representation of the embryogenesis of *L. stagnalis* modified after Morrill, showing the length of the embryo, and some of the morphological criteria and behavioural features, was used to determine different embryonic stages (see Supplementary Fig. 1). However, the exact time of embryogenesis depends on a number of conditions such as temperature, photoperiod, and ionic-composition of water, at the location where they are being raised. In this respect, the development from first cleavage (E0 stage) to embryos hatching (E100 stage) takes place approx. 14–15 days in our laboratory, similar to others (Marois and Croll 1991). To determine the dynamic of hatching rate of embryos caused by different progestogen exposures, the generalised additive modelling (GAM) (Hastie and Tibshirani 1986) was applied (see Supplementary Fig. 2). Development of the vehicle control and treated embryos was monitored every day until hatching using a Leica M205c stereomicroscope equipped with a DFC3000G (Leica) digital camera. Pictures were taken every day, starting with the cell proliferation, following the changes until the hatching.

### Behaviour tests

#### In embryos

Based on the findings of Voronezhskaya and Filla (Filla et al. 2009; Voronezhskaya et al. 1999): heartbeat, gliding by their foot on the inner surface of the egg capsule, and feeding activity (radula protrusion) were monitored from the E65, E85, and E95 stages, respectively, in both control and progestogens-treated groups. Following the abovementioned studies, the heartbeat and the radula protrusion (as fast behaviours) were counted for 2 min, while the number of circles performed by gliding embryos (as a slow behaviour) was monitored for 4 min by Leica M205c stereomicroscope. To make the results of heartbeat more comparable (to reduce standard deviation), the relative numbers were used in the case of all groups from 3 independent series.

#### In adults

Locomotion test—Snails from vehicle control and progestogen-treated groups were individually placed in an experimental tank (10 × 20 × 3 cm, (Salanki et al. 2003)) on the 21st day of the treatments. After acclimatisation for 10 min, the locomotion route of snails was marked continuously by a marker for 4 min. Digital photographs of each animal were taken by Nikon D5100 camera after the test. Based on individual pictures, the traces made by a single animal were measured (in cm) and analysed using the Mousotron8.2 freeware software (BlackSun, www.techspot.com/download).

Feeding test—Feeding behaviour was followed by placing the snails individually into a Petri dish filled with 20% sucrose solution, which evokes feeding activity, i.e. rhythmic opening–closing movements of the mouth (Kemenes et al. 1986). The feeding experiment was made on the 21st day of treatment. After 10-min acclimatisation, the evoked feeding rate was characterised by a counter for 2 min (the number of bites/2 min).

### Statistical analysis

Statistical analysis was performed using the OriginPro8 2015 software (OriginLab Corp., Northampton, Massachusetts, USA). Normality of the dataset was investigated using the Shapiro-Wilk test, homogeneity of variances between groups investigated using Levene’s statistic. For the analysis of hatching time, heart rate and feeding activity of embryos, two-way repeated-measure ANOVA was used to assess main effects of time, treatment, and time treatment interaction. This analysis was followed by one-way ANOVA and Scheffe’s post hoc test to identify significant differences between control and treatment groups within a given time point. Differences between the control and treated groups of gliding activity of embryos as well as locomotion and feeding activity of adults were analysed using one-way ANOVA with Scheffe’s post hoc test. Differences were considered statistically significant at *P* < 0.05 (*) and *P* < 0.01 (**). Error bars in the figures indicate mean ± standard error.

## Results

### Embryos

**Fig. 1:**
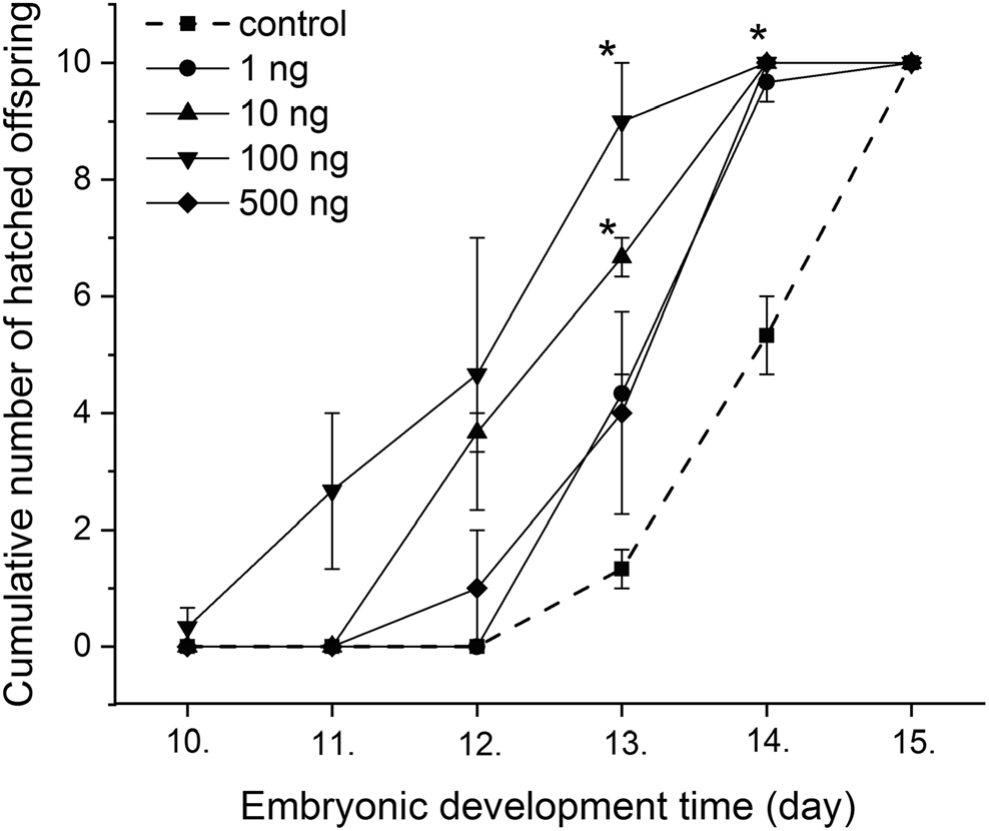
Embryonic development and hatching rate during the progestogen exposure. Dotted line indicates control. *P < 0.05, between control and treated groups. Error bars in the figures indicate mean ± s.e. *n* = 10 embryos/group/well/replicates

**Fig. 2:**
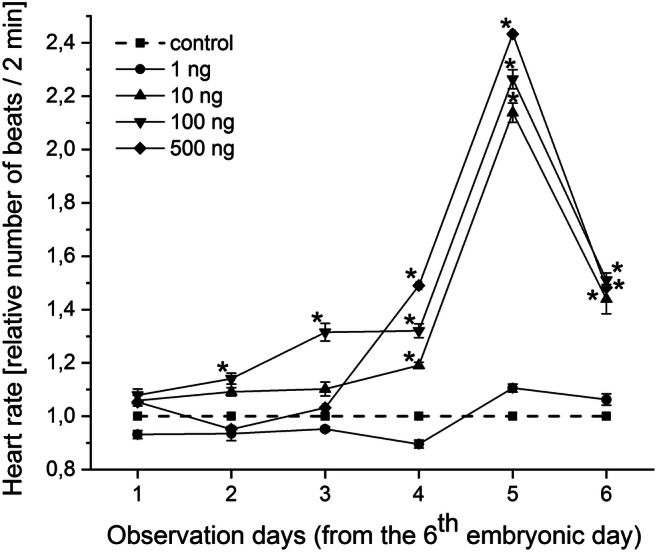
Relative heart rate of embryos from the 6th embryonic day. Number of heartbeats in the 2-min test period is shown. Interrupted line indicates control. Within a single observation day, *P < 0.05 between control and treated groups. Error bars in the figures indicate mean ± s.e. *n* = 10 embryos/group/well/replicates

**Fig. 3:**
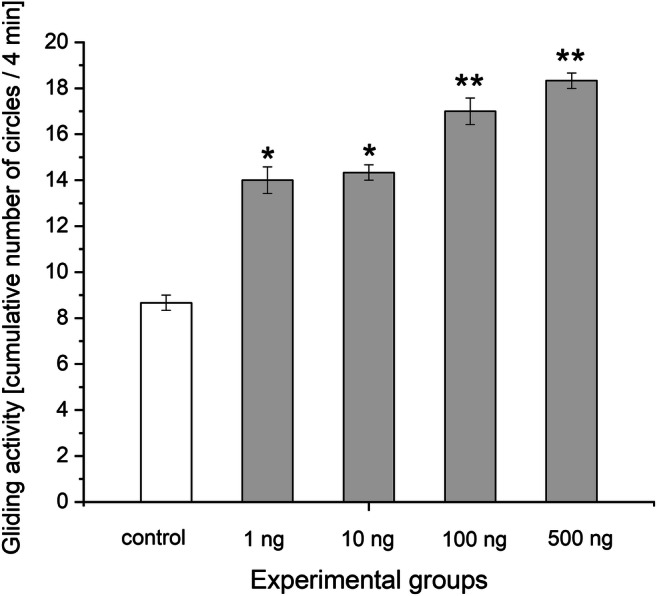
Gliding activity of embryos in different experimental groups. Cumulative number of circles performed by gliding embryos during 4-min time window is shown. The white column represents the control while the greys the treated groups. *P < 0.05, **P < 0.01 between control and progestoge-treated groups. Error bars in the figures indicate mean ± s.e. *n* = 10 embryos/group/well/replicates

**Fig. 4:**
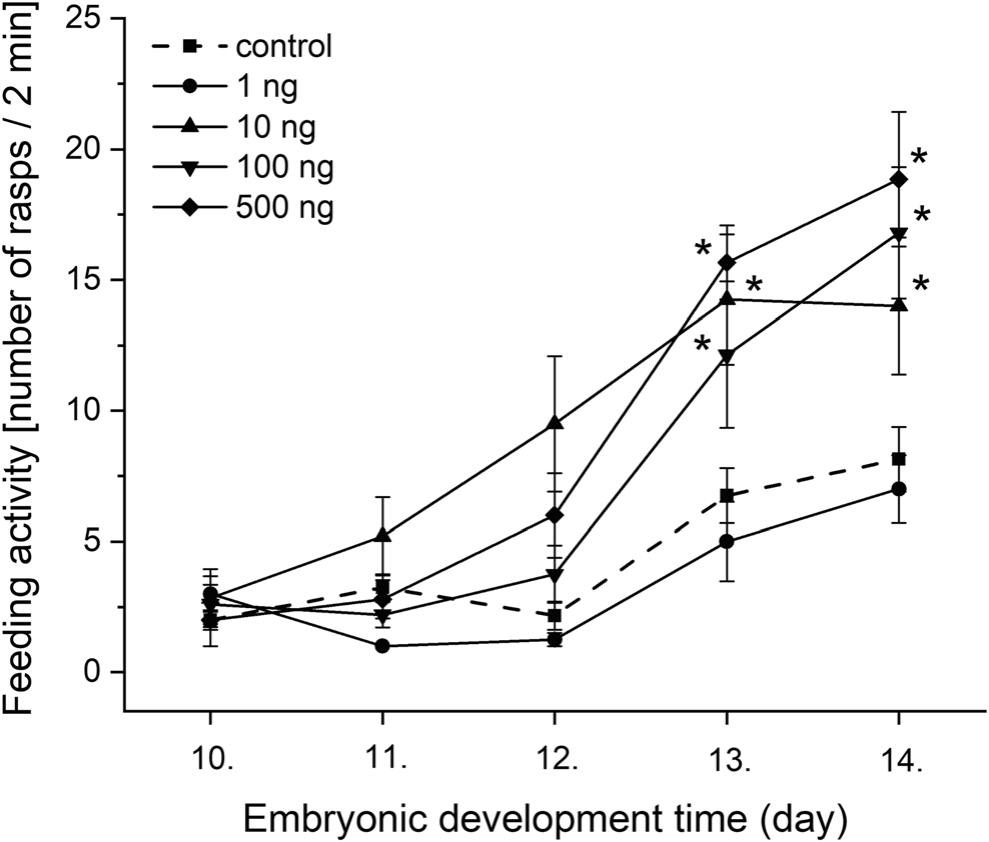
Feeding activity alterations of embryos observed on the different developmental days. Mean numbers of radula protrusion counted for 2 min are shown. Interrupted line indicates control. Within a single observation day, *P < 0.05 between control and treated groups. Error bars in the figures indicate mean ± s.e. *n* = 10 embryos/group/well/replicates

There was no observed lethality at any applied progestogen concentrations during the entire 15 days of the study period. The results of the effect of chronic progestogen exposure on the embryonic development of *L. stagnalis* are presented from the 10th embryonic day in Fig. 1. The progestogen exposure in all applied concentration caused a remarkable change in the hatching time of embryos. Two-way repeated-measure ANOVA revealed significant effects of time (observation day) [*F*(5, 60) = 209.49, *P* < 0.0001] and treatment [*F*(4, 60) = 20.92, *P* < 0.0001], and a significant time × treatment interaction [*F*(20, 60) = 4.26, *P* < 0.0001]. No significant difference was detected in the first 3 observation days between the control and treated groups. From the 13th embryonic day, the hatching rate significantly increased in the 10 and 100 ng L^−1^ -treated groups. The most of the embryos in the treated groups hatched to the 14th day (9/10, 10/10, 10/10, and 10/10 in 1, 10, 100, 500 ng L^−1^ -treated groups, respectively) showing a significant increase comparing to the control (average 5/10 on the 14th day). Alterations of the heart rate, followed from the 6th embryonic day (after the heart developed) for 6 days, are presented in Fig. 2 showing the relative heart rates of treated groups (standardised to original control values) to the control (standardised to themselves, *y* = 1). Two-way repeated-measure ANOVA revealed significant effects of time (observation day) [*F*(5, 60) = 155.54, *P* < 0.0001] and treatment [*F*(4, 60) = 81.02, *P* < 0.0001], and a significant time × treatment interaction [*F*(20, 60) = 11.67, *P* < 0.0001]. Further analysis with one-way ANOVA and post hoc test indicated that there was no significant difference in the heart rate between the control and 1 ng L^−1^ progestogen-treated groups in case of all time points (observation days). No significant difference was detected in the first 3 observation days between the control and 10, 500 ng L^−1^ groups; however, the embryos of the 100 ng L^−1^ -treated group started showing significantly higher heart rate on the 2nd and 3rd days. On the 4th–5th observation days, the heart rate of the embryos of 10, 100, and 500 ng L^−1^ -treated groups significantly increased with a maximum 2.1-, 2.2-, and 2.4-fold change, respectively. However, during the developmental progress, this exciting effect decreased to 1.5-fold change (still significant). Before hatching, embryonic heartbeat is known to start being similar to the lower postembryonic heartbeat, the shorter duration of embryonic life in the progestogen-exposed groups (see Fig. 1) can explain the lower fold change. Intracapsular locomotor activity was followed for 3 days while embryos performing an active gliding behavior from E80 to E90 (Fig. 3). During this period, the cumulative number of gliding activities significantly increased in all of the exposed groups. Alterations of the embryonic feeding activity, followed from the 10th embryonic day, are presented in Fig. 4. Two-way repeated-measure ANOVA revealed significant effects of time (embryonic day) [*F*(5, 60) = 18.17, *P* < 0.0001] and treatment [*F*(4, 60) = 3.78, *P* = 0.0062], but not significant time × treatment interaction [*F*(20, 60) = 1.03, *P* = 0.4238]. Further analysis with one-way ANOVA and post hoc test indicated that there was no significant difference between the control and 1 ng L^−1^ progestogen-treated groups during the whole observation period. The characteristic shape of the curve of 1 ng L^−1^ group follows that of control. No significant difference was detected in the first 3 observation days between the control and the 10, 100, and 500 ng L^−1^ groups. From the 13th embryonic day, the radula protrusion significantly increased in the 10, 100, and 500 ng L^−1^ treated groups.

Interesting observation that the investigated behavioural activities including heartbeat (Fig. 2), gliding (Fig. 3), and feeding activity (Fig. 4) consequently increased in the 10, 100, and 500 ng L^−1^ -treated groups.

### Adult snails

**Fig. 5:**
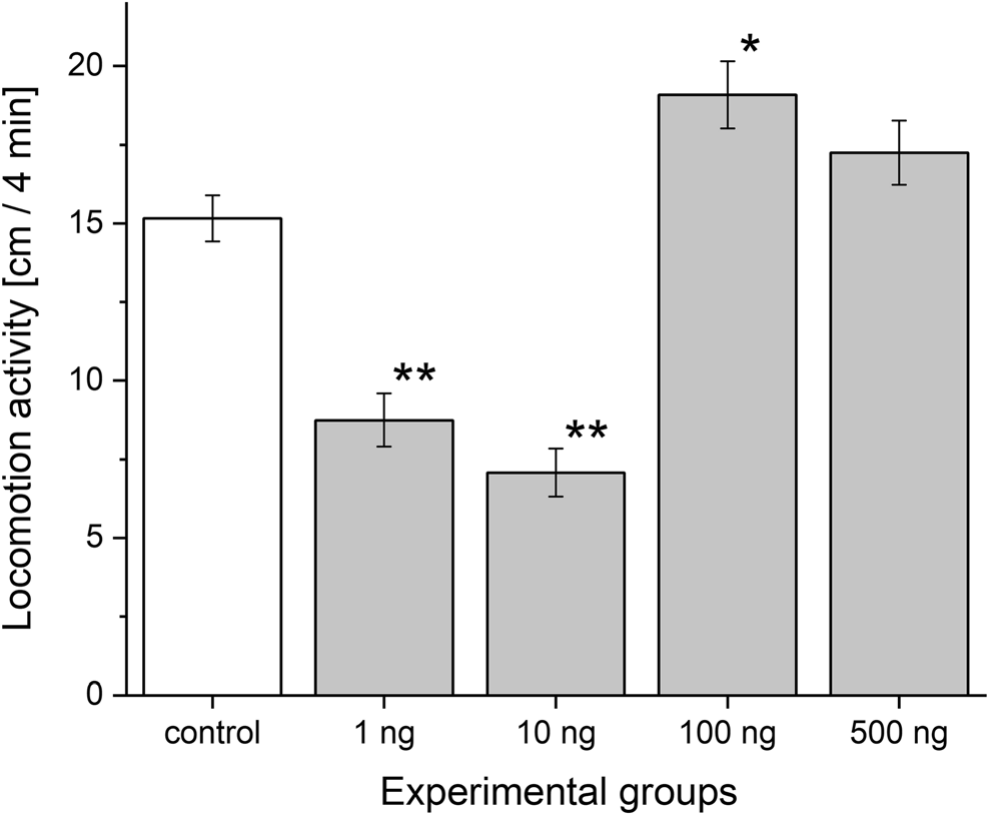
Locomotor activity in adult snails of experimental groups. Mean distances covered by snails during the 4-min test period are presented. The white column represents the control while the greys the treated groups. *P < 0.05, **P < 0.01 between control and progestogen-treated groups. Error bars in the figures indicate mean ± s.e. *n* = 12 adults/group/tank/replicates

**Fig. 6:**
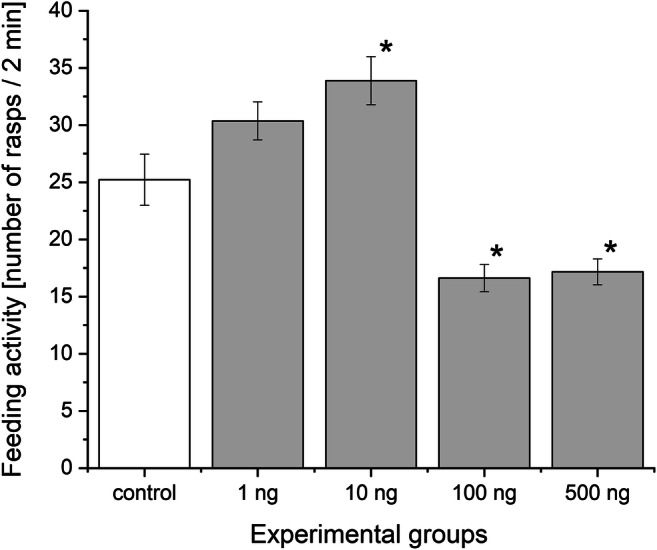
Feeding activity in adult snails of experimental groups. Mean numbers of rasp counted for 2 min are shown. The white column represents the control while the greys the treated groups. *P < 0.05 between control and progestogen-treated groups. Error bars in the figures indicate mean ± s.e. *n* = 12 adults/group/tank/replicates

Similar to the embryos, the chronic progestogen treatments caused marked effects in the adult animals. The spontaneous locomotor activity altered remarkably by the various concentration treatments (Fig. 5). Specimens in the 1 and 10 ng L^−1^ groups covered a shorter average distance (8.7 ± 0.84 and 7.1 ± 0.76 cm, respectively) representing a significantly decrease in the locomotion compared with the control group (15.2 ± 0.74 cm). However, the locomotion test showed a significant increase in the 100 ng L^−1^ -treated group (19.1 ± 1.07 cm) compared to the control one. In contrast, the 500 ng L^−1^ group did not show significant difference in locomotion activity (17.2 ± 1.01 cm). The results of the exposure on the feeding activity are shown in Fig. 6. Compared to the control (25.2 ± 2.25 number of bites), the number of rasp slightly increased in the 1 ng L^−1^ group (30.4 ± 1.67 number of bites, but this was not significant) and significantly increased in the 10 ng L^−1^ progestogen-treated animals (33.9 ± 2.10 number of bites). However, the 100 and 500 ng L^−1^ groups showed a significantly decreased rate (16.6 ± 1.19 and 17.1 ± 1.14 number of bites, respectively) in the feeding activity. According to our findings, the progestogen exposure affected not only the embryos but also the adult specimens. The alterations of these adult behaviours showed a biphasic response; however, they changed approximately in the opposite way.

## Discussion

### Environmental relevance of experimental arrangement

To place our findings in such type of ecotoxicological studies and to evaluate their environmental relevance, we summarised the available international scientific data of PRG, DRO, GES, and LNG identified in different watercourses (Table 1). The concentration ranges of these compounds in ng L^−1^ are as follows: 0.06–9330.00, 0.20–170.00, 0.61–8.3, and 0.26–4.30 for PRG, DRO, GES, and LNG, respectively. Compared to these, we applied 1, 10, 100, and 500 ng L^−1^ progestogen concentrations for the exposure of *L. stagnalis* that can be definitely considered as environmentally relevant values. Following the experimental design of our previous study (Zrinyi et al. 2017), in which the results of recovery measurements (applying an HPLC–MS method by Avar et al. 2016) indicated that the actual concentrations were always ≥ 80% of the nominal concentration in experimental tanks even after 1 week, in the present study, the water and progestogens were renewed only weekly in case of adults. Using progestogens in mixture and in environmentally relevant concentrations, as they are present in the natural habitat (Guzel et al. 2019), for our controlled laboratory investigations, we made an effort to mimic the realistic environmental situation. We think that this approach was met in case of adult specimens but not fully in case of embryos.

**Table 1:** Measured environmental concentration (MEC) and summarized concentration range (italic values) of different progestogen residues in water samples. *PRG* progesterone, *LNG* levonorgestrel, *GES* gestodene, *DRO* drospirenone, *HPLC-MS/MS* high-performance liquid chromatography-tandem mass spectrometry, *UHPLC*-*MS/MS* ultra-high performance liquid chromatography-tandem mass spectrometry, *RRLC*-*MS/MS* rapid resolution liquid chromatography-tandem mass spectrometry, *SFC-MS/MS* supercritical fluid chromatography-tandem mass spectrometry, *GC-MS* gas chromatography-mass spectrometry, *ELISA* enzyme-linked immunosorbent assay

Progestogens	Watercourse name	MEC ng L^−1^	Analytical methods	References
PRG	Catchment area of Lake Balaton, Hungary	0.23–13.67	HPLC-MS/MS	Avar et al. 2016
	Catchment area of Lake Balaton, Hungary	0.60–2.30	SFC-MS/MS	Maasz et al. 2019
	Surface water, Rio de Janeiro, Brazil	0.51–47.20	LC-MS/MS	Kuster et al. 2009
	River Lee, Ireland	6.00	GC-MS	Aherne et al. 1984
	Paper mill effluent, Fenholloway River, USA	< 10.00	HPLC-MS/MS	Jenkins et al. 2003
	Urban Rivers, Bejing, China	26.00	LC-MS/MS	Chang et al. 2009
	Snowmelt runoff, Wisconsin, USA	375.00	HPLC-MS/MS	DeQuattro et al. 2012
	Grazing Rangeland surface water, California, USA	27.00	GC-MS/MS	Kolodziej and Sedlak 2007
	Surface water of agricultural, Pennsylvania, USA	7.35–11.81	GC-MS	Velicu and Suri 2009
	River Llobregat and drinking water, Barcelona, Spain	≤ 1.39	LC-MS/MS	Kuster et al. 2008
	Surface and ground water, French	1.70–4.10	LC-MS/MS (ESI)	Vulliet et al. 2008
	Surface and drinking water, Japan	0.06–0.09	LC–MS/MS	Chang et al. 2008
	Surface and drinking water, Conghua, China	1.20–2.50	UHPLC-MS/MS)	Liu et al. 2014
	Surface and drinking water, Baden, Switzerland	4.00–10.00	LC-MS/MS	Ammann et al. 2014
	River Danshui upstream Guandong, China	0.50 ± 0.10	RRLC–MS/MS	Liu et al. 2011b
	River Danshui downstream, Guandong, China	2.50 ± 0.10	RRLC–MS/MS	Liu et al. 2011b
	River Piracicaba, Brazil	0.58	LC-ESI-MS/MS	Torres et al. 2015
	Surface water, USA	0.199	GC-MS	Kolpin et al. 2002
	Domestic WWTP effluent, Belgium	2.50 ± 0.70	GC-MS/MS	Pauwels et al. 2008
	WWTP effluent, Japan	0.31–0.37	LC–MS/MS	Chang et al. 2008
	WWTP effluent, Bohai, China	0.80–2.30	RRLC-MS/MS	Liu et al. 2012
	WWTP effluent	2.90	LC-MS/MS	Yost et al. 2014
	WWTP effluent, Beijing, China	6.00	UPLC-MS/MS	Fan et al. 2011
	Domestic WWTP influent, Belgium	4.80–33.00	GC-MS/MS	Pauwels et al. 2008
	WWTP influent, Japan	3.10–10.00	LC–MS/MS	Chang et al. 2008
	WWTP influent, Bejing, China	66.00 ± 36.00	LC–MS/MS	Chang et al. 2011
	WWTP influent, Huiyang, Guangdong, China	6.10 ± 0.30	RRLC–MS/MS	Liu et al. 2011b
	WWTP influent, Meihu, Guangdong, China	5.40 ± 0.60	RRLC–MS/MS	Liu et al. 2011b
	WWTP influent, Bohai, China	38.00–108.00	RRLC-MS/MS	Liu et al. 2012
	WWTP influent	10.10	LC-MS/MS	Yost et al. 2014
	WWTP influent, Beijing, China	57.00	UPLC-MS/MS	Fan et al. 2011
	WWTP influent, Baden, Switzerland	4.15	LC-MS/MS	Ammann et al. 2014
	Animal farm waste water, Bohai lagoons, China	56.70–2470.00	RRLC-MS/MS	Liu et al. 2012
	Animal farm waste water, lagoons, China	29.00–11.90	RRLC-MS/MS	Liu et al. 2012
	Animal farm waste water, lagoons, Jiangmen, China	5024.00	UHPLC-MS/MS	Liu et al. 2014
	Animal farm waste water, lagoons	186.00–1430.00	LC-MS/MS	Yost et al. 2014
	Animal farm waste water, lagoons, Colorado, Denver	< 7.00–98.90	GC-MS/MS	Yang et al. 2012
	Animal farm (A) waste water, lagoons, China	1.70–9330.00	UHPLC-MS/MS	Liu et al. 2015
	Animal farm (B) waste water, lagoons, China	2.31–5402.00	UHPLC-MS/MS	Liu et al. 2015
Concentration range of PRG:		*0.06–9330.00*		
LNG	Catchment area of Lake Balaton, Hungary	0.85–3.40	HPLC-MS/MS	Avar et al. 2016
	Catchment area of Lake Balaton, Hungary	1.90–49.40	SFC-MS/MS	Maasz et al. 2019
	Surface and ground water, French	5.30–11.00	LC-MS/MS	Vulliet et al. 2008
	Mean surface waters, Rhône-Alpes region, French	3.60	LC-MS/MS	Vulliet and Cren-Olivé 2011
	River water, Malaysia	38.00	LC-MS/MS	Al-Odaini et al. 2010
	Rivers Anoia and Cardener, Catalonia, Spain	< 0.20–4.00	LC-MS	Petrovic et al. 2002
	WWTP effluent, Catalonia, Spain	< 0.20–4.00	LC-DAD-MS	Lopez de Alda et al. 2002
	WWTP effluent, River Seine, French	< 2.50–7.20	GC-MS	Labadie and Budzinski 2005
	WWTP effluent, River Jalle d'Eysines, French	< 2.00–5.00	GC-MS	Labadie and Budzinski 2005
	WWTP effluent, area of Lyon, French	0.90–17.90	LC-MS	Vulliet et al. 2007
	WWTP effluent, China	1.10	HPLC	Pu et al. 2008
	WWTP effluent, China	1.30	ELISA	Pu et al. 2008
	WWTP effluent, Montreal,Canada	30.00	LC-MS/MS	Viglino et al. 2008
	WWTP effluent, River Funan Chengdu, China	8.10	HPLC	Qiao et al. 2009
	WWTP influent, River Funan Chengdu, China	74.30	HPLC	Qiao et al. 2009
	WWTP influent, Montreal,Canada	150.00–170.00	LC-MS/MS	Viglino et al. 2008
	WWTP influent, China	6.50	ELISA	Pu et al. 2008
	WWTP influent, China	5.60	HPLC	Pu et al. 2008
	WWTP influent, Spain	< 0.20–16.10	LC-MS	Petrovic et al. 2002
	WWTP influent, Catalonia, Spain	< 0.20–16.00	LC-DAD-MS	Lopez de Alda et al. 2002
	WWTP influent, Bejing, China	4.90 ± 1.20	LC-MS/MS	Chang et al. 2011
Concentration range of LNG		*0.20–170.00*		
GES	River Danube, Hungary	3.60	LC-MS/MS	Neale et al. 2015
	WWTP effluent, Beijing, China	0.61–8.30	UHPLC-MS/MS	Shen et al. 2018
Concentration range of GES		*0.61–8.30*		
DRO	Catchment area of Lake Balaton, Hungary	*0.26–4.30*	HPLC-MS/MS	Avar et al. 2016

Pond snail embryos have been the subject of many studies, but in most cases, toxic effects, such as hatching, were determined for intact egg masses (Gomot 1998; Khangarot and Das 2010; Das and Khangarot 2011). However, as also presented in a previous study which compared the sensitivity of isolated eggs and intact egg masses of pond snail *Radix auricularia* to cadmium (Liu et al. 2013), the gelatin matrix around eggs of egg masses has evolved to protect them against threats from the environment during their development. Therefore, it limits the sensitivity of snail embryos to some extent. Furthermore, eggs frequently infected by parasites in the field (or even in the laboratory in some cases) can be discarded by the isolation procedure. Hence, the isolated eggs seem better suitable for toxic assays and risk assessment since they lower the individual differences between developing eggs. Moreover, similar to previous studies (Marois and Croll 1991; Voronezhskaya et al. 1999; Filla et al. 2009), usage of isolated eggs embryos has even a technical reason: ensuring much greater synchrony in hatching, the appropriate tracking of behaviours, and more standardised and reproducible experiments.

### Progestogens-induced alterations in *L. stagnalis*

In our previous study, we presented that parental progestogen exposure caused alterations in the intracapsular development and metabolomics composition in the examined early phase of embryonic development of *L. stagnalis* (Zrinyi et al. 2017). In the present study, we extended this previous investigation, exposed the embryos and followed the alterations during the whole embryonic development to get more information about the mode of action of different progestogen concentrations (1, 10, 100, and 500 ng L^−1^). The parental exposure did not influence the hatching time of embryos (Zrinyi et al. 2017); however, the direct exposure of isolated eggs in the 10, 100, 500 ng L^−1^ -treated groups resulted in a quicker hatching rate (Fig. 1). External environmental stimuli including active drug residues are known to influence the embryonic development. One such potential pathway could be the energy budget of the developing embryos; our previous results, as there is a significantly elevated hexose utilisation in the embryos and elevated adenylate energy charge in the egg albumen (Zrinyi et al. 2017), support this theory. It is possible (though speculative) that the elevated energy utilisation induced by progestogens is present during the whole embryonic development which could explain the observed acceleration of the embryonal behaviour activities (heartbeat, gliding, and radula protrusion). However, to determine the exact underlying mechanisms, further investigations are required with energy-based approaches such as dynamic energy budget theory (Zonneveld and Kooijman 1989; Kooijman 2000; Ducrot et al. 2010). In case of the hatching rate of embryos as well as the feeding activity of adult specimens, the dose-response phenomenon can be identified as a hormesis response (Calabrese and Baldwin 2003). The opposite change of feeding and locomotor activity of adult specimens can be explained with previous results as the activation of some kind of motor activity simultaneously suppresses ongoing feeding ingestion behaviour, even in the presence of food (Pirger et al. 2014). Furthermore, it was discovered that the locomotor and feeding autonomous circuits underlying the execution of these two opposing behaviours are connected via a single type of interneuron, the pleurobuccal (PlB) cell, functioning as a switch (Pirger et al. 2020).

In general, there is a diverse literature on the reproductive and developmental effects as well as the induced behavioural responses of potential endocrine disruptive PhACs including progestogens in invertebrates. Furthermore, there is a long-standing debate about whether, or not, natural vertebrate and synthetic sex steroid residues occurring in the environment can affect the neuroendocrine system and physiological processes of molluscan species (Alzieu 2000; Amorim et al. 2019; Fodor et al. 2020b; Matthiessen and Gibbs 1998; Scott 2012, 2013, 2018; Tran et al. 2019). Previous studies demonstrated that three key steps—cholesterol side-chain cleavage, 17-hydroxylation, and aromatisation—of the classical vertebrate steroid biosynthetic pathway are either absent, or occur very weakly, in molluscs (reviewed in Fodor et al. 2020b; Scott 2012). Most importantly, the homologues of the enzymes that catalyse the first and third of these reactions in vertebrates, as well as the functional sex steroid receptors, have so far not been found in molluscan genomes (Fodor et al. 2020b). Yet, several papers presented that molluscs do seem affected by sex steroids occurring in the surface waters, though most of the bioassay information has been contradicted (reviewed in Scott 2013). Focusing on progestogens, progesterone was shown to affect gametogenesis in the snail *Helix pomatia* (Csaba and Bierbauer 1979) and in the scallop *Mizuopecten yessoensis* (Varaksina et al. 1992), to induce vitellogenesis, oocyte proliferation, and spermatozoa activation in the octopus *Octopus vulgaris* (Di Cristo et al. 2008; Tosti et al. 2001), as well as to induce *in vitro* gamete release in the scallop *Placoplecten magellanicus* (Wang and Croll 2003). Besides, though not a progestogen, another endocrine disruptors such as the antiandrogenic (fungicide) vinclozolin was shown to affect some reproductive parameters of *L. stagnalis* after 21-day long exposure in μg L^−1^ concentrations (Giusti et al. 2014). Based on all of these data, though most likely via non-specific interactions (e.g. with receptors for other compounds) (Fodor et al. 2020b; Scott 2012), the embryos and adult specimens of *L. stagnalis* seem to be sensitive to progestogen contaminations that occur in their natural habitat, but the exact mode of action underlying the ecotoxic effects of these synthetic PhACs remains to be determined. In order to reveal the possible underlying cellular mechanisms, we investigated the changes of four relevant key molecules in the CNS: DJ-1, cAMP responsive element-binding protein (CREB), p38alpha, and c-Jun N-terminal kinase 1 (JNK1). These molecules are identified in *L. stagnalis* (see Supplementary information). DJ-1 was previously determined as a potential biomarker for environmental progestogen exposure in fish (Maasz et al. 2017). CREB is known to be involved also in different sex steroid signal pathways (Lazennec et al. 2001). p38alpha and JNK1 are considered as stress-activated protein kinases that participate in the cellular response to metabolic and other (environmental) stress conditions such as hormones (Bengal et al. 2020). Furthermore, p38 expression was previously shown to increase in the vertebrate CNS after progestogen treatment (Blackshear et al. 2017). We have found that these molecules show significant quantitative changes during the progestogen exposure (Supplementary Fig. 3). Besides, we would like to highlight that this study does not focus on what the molecular background may be. We just readily accept that progestogens are present in the natural habitat of *L. stagnalis* and investigated whether their presence can cause any detectable changes in the different embryonic and adult behaviours.

## Conclusions

The concentrations of progestogens in the environment vary widely, from a few ng L^−1^ to a few hundred ng L^−1^ in average. Based on our findings, these progestogens, which occur also in the natural habitat of *L. stagnalis*, can affect the embryos and adult specimens even at average environmental concertation (~ 10 ng L^−1^). We observed several induced alterations in the different behaviours such as in the embryonic development time, heart rate, feeding, and gliding activities of embryos as well as in the feeding and locomotion activity of adult specimens. These non-reproductive effects of progestogens were not reported previously on molluscan species. The ecological relevance of our findings was adequate in case of adults but this was moderate in embryos due to applied experimental approach. Our results are consistent with previous data as embryos and adult specimens of *L. stagnalis* are sensitive to human PhAC residues including progestogens, even at low concentration. However, without identified functional steroid receptors, the molecular mechanisms underlying the physiological and behavioural effects are unknown at present. Although we investigated the potential role of four key molecules, the exact mode of action needs to be determined for understanding these drugs’ effects on snails including *L. stagnalis*.

## Supplementary Information

ESM 1 (DOCX 1142 kb)

## Data Availability

All relevant data are within the manuscript and available from the corresponding author upon request.
