# Supplementary material for: Progestogen-induced alterations and their ecological relevance in different embryonic and adult behaviours of an invertebrate model species, the great pond snail (Lymnaea stagnalis)
Source: Environ Sci Pollut Res Int. 2020 Dec 21;28(42):59391–402. doi: 10.1007/s11356-020-12094-z (PMC8542004; doi:10.1007/s11356-020-12094-z)
Supplement: Supplementary file 1 — (DOCX 1142 kb) [file 11356_2020_12094_MOESM1_ESM.docx]

**Supplementary information**

**Progestogen-induced alterations and their ecological relevance in different embryonic and adult behaviours of an invertebrate model species, the great pond snail (*Lymnaea stagnalis*).**

^1,2^Reka Svigruha, ^2^Istvan Fodor, ^1^Judit Padisak, ^2^Zsolt Pirger*

^1^Department of Limnology, University of Pannonia, 8200 Veszprém, Hungary

^2^NAP Adaptive Neuroethology Research Group, Department of Experimental Zoology, Balaton Limnological Institute, Centre for Ecological Research, 8237 Tihany, Hungary

*****Corresponding author; E-mail: [pirger.zsolt@okologia.mta.hu](mailto:pirger.zsolt@okologia.mta.hu) (Z Pirger)

**Supplementary Fig. 1** – Schematic representation of the embryogenesis of *L. stagnalis* modified after Morrill (1982), showing the length of the embryo, and some of the morphological criteria and behavioural features used to determine different embryonic stages. Grey arrows indicate the embryonic stages in which the different behavioural activities were started to be monitored: 65% (heart), 85% (gliding), and 95% (radula protrusion). White arrowhead shows the radula of embryo before hatching while black interrupted circle represents the heart of embryo during the developmental stage of E65.

**
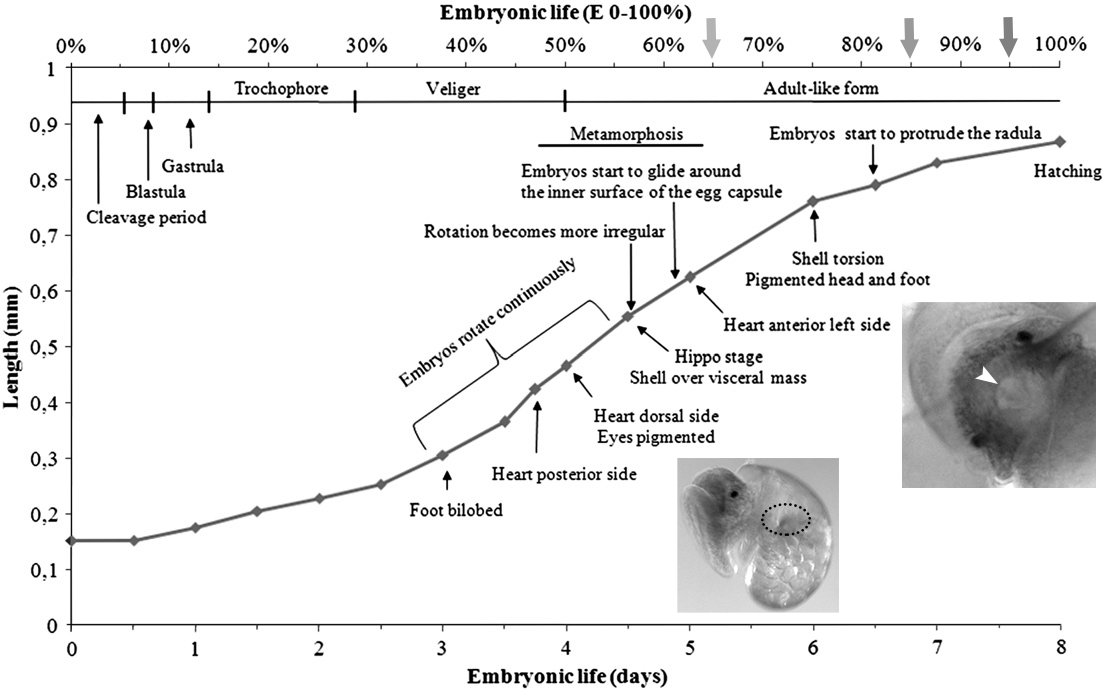
**

**Supplementary Fig. 2 -** Cumulative hatching by unit of time during the progestogen exposure based on GAM models. Blue line the model prediction, while grey regions are 95% confidence intervals.

**
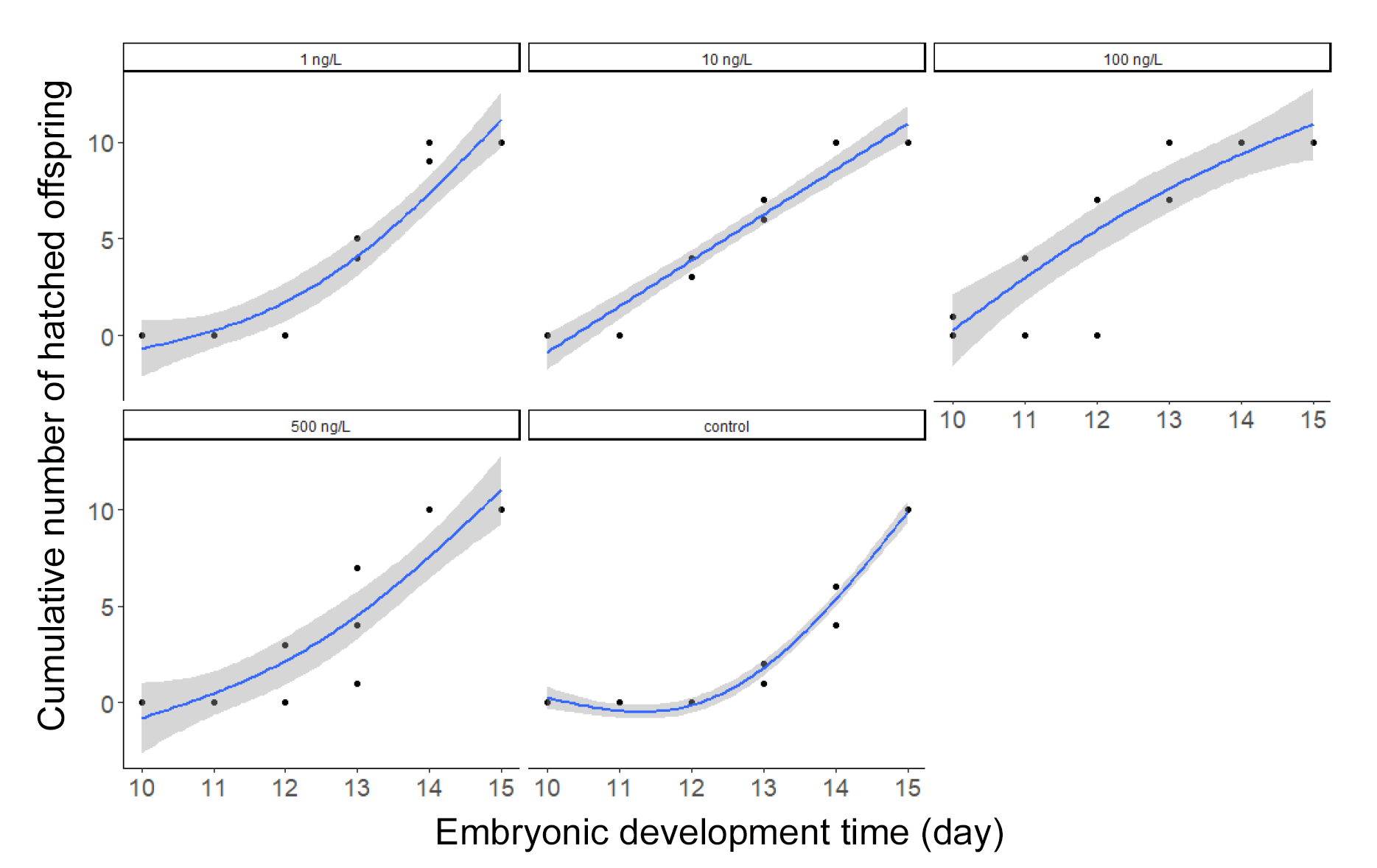
**

**Supplementary Fig. 3 -** Quantitative analysis of subcellular proteins of *L. stagnalis*. Representative sandwich ELISA (A) and phospho-MAPK array (B-D) showing key proteins concentration in the CNS of control and 10 ng/L treated animal groups following 1 week and 3 weeks progestogen exposure. A – Vertical axis shows the concentration of protein deglycase DJ-1 [ng/mg tissue], the horizontal axis illustrates different groups (n=6/group) and time points. The DJ-1 concentration was significantly decreased in the treated group compared to the control after the 1^st^ week [t(10)= 6.224, P<0.001,***) but significantly increased after the 3^rd^ week [t(10)= -3,586, P<0.01,**). B, C and D – Vertical axis shows the normalized data that are expressed as mean of pixel intensity ± S.E.M, the horizontal axis illustrates different groups (n=6/group) and time points. Both of the concentration of cAMP responsive element binding protein (CREB, B) and p38alpha mitogen-activated protein kinase (p38alpha, C) significantly increased in the treated group compared to the control after the 1^st^ week [t(10)= -2,485, P<0.05,*; t(10)= -2,836, P<0.05,* respectively] but significantly increased after the 3^rd^ week [t(10)= 2,464, P<0.05,*; t(10)= 3,627, P<0.05,* respectively]. The concentration of c-Jun N-terminal kinase 1 (JNK1) did not changed significantly after the 1^st^ week but significantly decreased in the treated group compared to the control after the 3^rd^ week [t(10)= 7,51131, P<0.05,*].


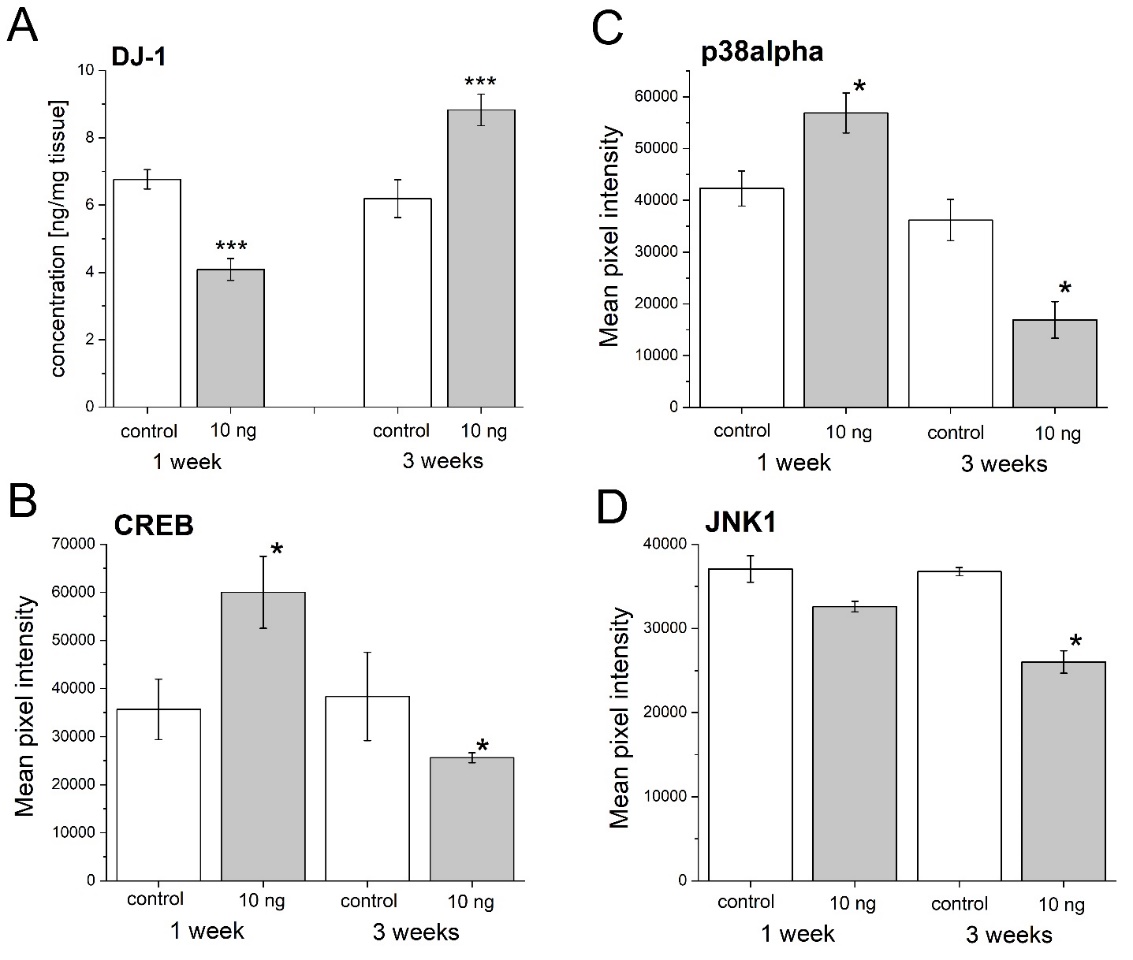


**Sandwich ELISA**

The whole CNS of control (n=6) and 10 ng L^-1^ progestogen mixture treated (n=6) snails was used for ELISA. Samples were homogenized in reagent diluent (2.5 mg tissue/100 μL) then centrifuged (Heraeus Biofuge Pico, Thermo Fisher Scientific) at 8000 g for 10 min at 4°C. The supernatants were collected and transferred to ultra-high recovery Eppendorf tubes.

Special 96-well micro plates (DJ-1/PARK7 DuoSet ELISA Kit; R&D systems) were coated with 100 µL/well of capture antibody at 0.8 µg/ml in PBS solution at room temperature overnight. The plates were washed three times with buffer (0.05% Tween 20 in PBS, pH 7.2-7.4). Blocking was performed with reagent diluent (300 µL/well, 1% BSA in PBS, pH 7.2-7.4) and incubated at room temperature for 1 h. After washing, 100 μL of the protein deglycase DJ-1 standards (0.313; 0.625; 1.25; 2.5; 5 ng/mL) were added to each well along with the 100 μL/well snail antigen samples. Method control was made with capture and detection antibody containing reagent diluent. Triplicates made on the plate for each sample were incubated at room temperature for 2 h. The washing protocol was repeated. Then 45 ng/mL detection antibody in reagent diluent (100 µL/well) was added and incubated at room temperature for 2 h, followed by an additional washing step. HRP (horseradish peroxidase) conjugated detection antibody was added 1:40 in reagent diluent (100 µl/well) and incubated at room temperature for 20 min in dark, according to the protocol. The bound HRP conjugate was detected by adding 3,3′,5,5′-Tetramethylbenzidine (TMB)-one ready to use (Sigma Aldrich, 100 µL/well), and incubated in dark place at room temperature for 20 min. The presence of immune-complex was detected by a blue-colour development and the enzymatic reaction was stopped by additional 100 µL/well 2N H_2_SO_4_. Finally, the optical density was analysed with a micro plate reader (PerkinElmer, Victor3 1420 multilabel counter) at 450 nm, and corrected subtraction was used at 550nm for avoiding imperfections in the plate.

DJ-1 protein was sequentially identified in *L. stagnalis* (#MT153192.1) and demonstrated to be highly conservative by our research group (Fodor et al., 2020). Furthermore, DJ-1/PARK7 DuoSet ELISA Kit has already been successfully applied for quantitative analysis of DJ-1 in *L. stagnalis* (Maasz et al., 2017).

**MAPK Array Analysis**

For investigating the effect of progestogen exposure on expression of various MAPK pathway proteins in snail CNS homogenates, Human Phospho-MAPK Array Kit from R&D System (Biomedica Hung., Budapest, Hungary) was used. Whole CNS from control (n=6) and progestogen treated (n=6) adult snails was removed and processed for MAPK array analysis. The array was performed as described by the manufacturer. The kit contains all necessary content. Briefly, after blocking the membranes for 1 h and adding the reconstituted detection antibody cocktail for another 1 h at room temperature, membranes were incubated with sample/antibody mixture at 2-8 °C overnight. After washing, horseradish peroxidase-conjugated streptavidin was added for 30 min, then membranes were exposed to a chemiluminescent reagent. Array data were analysed by ImageJ software. Based on previous (Sadamoto et al., 2004) and our present sequence data from *L. stagnalis* (see below), CREB (#BAC20140.1), p38alpha (#MW322711), and JNK1 (#MW322712) show high similarity to the relevant human sequences, therefore the Human Phospho-MAPK Array Kit is suitable for the analysis of their protein concentrations.

**Statistical analysis**

Statistical analysis was performed using the OriginPro® 2018 software (OriginLab Corp., Northampton, Massachusetts, USA). Differences in the amount of the subcellular *L. stagnalis* proteins were analysed using the independent samples t-test.

**Experimental animals, nucleotide sequencing, and bioinformatics**

For nucleotide sequencing, the whole CNS was dissected from the snails (n=10) and homogenized using a TissueLyser LT (QIAGEN) in TRI reagent (#93289, Sigma-Aldrich). RNA was isolated with Direct-zol^TM^ RNA MiniPrep (#R2050, Zymo Research) following the instructions of manufacturer. The RNA was quantified by Qubit BR RNA Kit (#Q10211, ThermoFisher) and the quality was checked on Agilent Bioanalyzer 2100 using RNA 6000 Nano Kit (#5067-1511, Agilent).

Nanopore sequencing was used to identify evolutionary conserved signal transduction molecules. The library was prepared using cDNA-PCR Kit (#SQK-PCS108, Oxford Nanopore Technologies) according to the description of manufacturer. The sample was sequenced on a MinION device with R9.4.1 flowcells (#FLO-MIN106). Base calling was performed using Guppy v3.2.2 software. Relevant human sequences (p38alpha and JNK1) from NCBI database (#NP_001306.1; #P45983.2) were used as search queries. Adapters were trimmed with Porechop v0.2.4 (Wick, 2018), moreover sequences with internal adapters, which were indicating chimera reads, were also splitted with Porechop. Reads were assembled with CLC Genomimcs Workbench v12.0.3 software *de novo* pipeline (QIAGEN). Consensus sequence was called and manually corrected also within CLC Genomics Workbench. For verification and sequence correction, the findings were compared with virtual cDNA sequences extracted from the unannotated genomic data (generated by Illumina sequencing) to which we have access as part of the *L. stagnalis* genome consortium (genome publication in preparation). The identified sequences were submitted to the NCBI Nucleotide database (#MW322711; #MW322712). Conserved domain search using NCBI CDD/SPARCLE (Marchler-Bauer et al., 2017; Lu et al., 2020) was performed to check if the key regions are present in the deduced protein sequences.

**Nucleotide and deduced protein sequences with conserved domains of *L. stagnalis* findings**

**>Lymnaea stagnalis_p38 alpha homolog_mRNA (#MW322711)**

AAAATTATTCCGTATACCGGAAGTAATACACACAACTTCGGGGAAAGACGACAATATTTTCAGGCTACAAATCGAGATATTCTTGCGTCAAATTTAGTATCTGTTACTCTGCTGTGCAGGATATTTCTTGGATTGGCATTTAGTGCATGGACTAATGGCGGATACTGCGCAACGCAAACCAGGGCTTGTGACAGTGGAATTAAATAAAACGATATGGGAGGTGCCCAGTCGATACAAAATCCAGAATCCTGTTGGAATTGGGGCATACGGTCAAGTCGTATCTGCAACTGACAACCTATTATGCACTCGAGTGGCCATTAAGAAACTTGCCCGCCCTTTTCAAACTGCCATCCATGCCAAAAGAACTTACAGAGAACTACGTATGTTAAGGCATATGAACCATGAGAATGTGATTGATCTAATTGATGTGTTCACACCAACAGTTACCCTGCAGGATTTTACAGATGTATACCTGGTGACTCCATTGATGGGAGCTGATCTCAATAACATACTCAAAACACAAAGGCTTAGCGATGACCATGTACAGTTTCTTGTTTACCAAATTCTCAGAGGTTTAAAGTACATTCATTCAGCTGGAATTTTACACAGGGACCTCAAGCCAAGTAATATTGCAGTGAATGAAGACTGTGAATTGAGGATTTTAGATTTTGGTCTAGCTCGTCTTACTGATGAAGAGATGACAGGTTATGTAGCCACAAGATGGTACAGGGCCCCAGAGATTATGCTTAACTGGATGCACTACAATCAGACAGTGGATATCTGGTCAGTTGGCTGTATTATGGCAGAAATGTTAACAGGACGACCCCTCTTTCCAGGCAGTGACCACATCGACCAGCTGACAAGAATCTTAAATCTTGTAGGAACTCCAAACGATGAACTCATGGAGGAAATCAAAAGCCAAGATGCAAAACTGTTCATCAAATCTCTACCTGTGATGACTCGTAAAGACTTCAAACAAGTTTTTGCTGGATCCAACCCACTAGCCATTGATCTACTGGAGAAAATGCTGGATCTCAACACAAAGTCGAGACTCAATGCTACACAAGCCCTCGCACATGAGTATCTAAAACAGTATGCAGATCCTGCTGACGAACCAGTGTCTGAGAAATATGACATGACTTTTGAAGATTATGACCTTAGTATCAATGAGTGGAAACAGCTTGTATTTGAAGAAATAGAAAAATTTCAAGCGGCCCATCAACTCTCATAGCACGTCCATATCCTGAAGCTGTATGGAAGTAGGTTCGTTCAGAGGACTATTTAAAATCACAGCACTGAGCAAAGGGCAACTCTGTTTTTTTTTATTGTAGAGAAATAACCCTGAAGGTAAATTTAACTAAGAAGATCTTGTGTTGAAATGAAAAATTTTAAAAATCATTAGAATGGTATGAAAATAACCTCGCTGTTTATGGGTAATTTTTCACTTTTTTTATAATTAAATCATAACATTCTGTATTTTTTAATTGAATTAAGGTAAATAAGTTTTGTGGTCATGTGTGTCCACTATTCACCGCATTCATTATGACCTAGTGACGAATCCCTTCTTATGGTTTGAACATTTTTGTTCCAGTTTTGTTGATTGATTCGAAGCTTTGTAATCAACTGTTGCGAACATGTTAAATAATTGATGTCTTGTACTAACTTGTTGGTTTTCTTGTTGTTTTTTGTTTAAAAATATTACTATATTTTTGGGTTTTTTTTACATGCGCAGTGCAGCATGTCTATTCATCTTGTAAAATGGACAGTAAACACATAAATGCATGATTGAATTGTGACGTGAATCATGAAAGAAAAGAAAATATTGACAAAACCGTATTTCAAATTTAACAATTTCAATTTTTAAAAAGCTTAATTACGCTCTTCATGTGAATTTTTAAAAAAAAATTGTTATTAAAATTAAAATGAAGGAAAGCTTAACAGACTTTCACATTACCTTTTATACACTGCTTTAACTTTTCTAGCAATGCCTCTATCAATGACTCATTCTTACGTGTGCTATGGTCAGAAAATGATGCATGAATTGCAGCTAGGGGCAACTACTTACGTTATGAGAATTATAGAATTATGTTTCAAAATAAGTTCCTCTGTCATGAGATTAATTTATTTAAAAGCTGAACTTTGATGTATTTTTAAAATCCTTTATATTAACTTCGCTATTGTTCATTGCTGGAAAAAATCTTGAGACAGCTAATTGACCCACTTCTTGTCAACCTGTTGAGCTGAAACTTGGCACTCATTGCTGATGACAAGACATTCTGTCTAATTTTCAACCCTAACCCCCAAAAATCAAATTTTTCGGTTTTTCAAGGGGAAAATGAAGGAAATATGATGCTACTGACTTCAAGAAATAAAATCCCAGATAACTGCACTAAATGAGATTCTTTTTCAAAGAATCTATGGCAAGTGCGTTTGGAGCGCATTATTTTCTATTGTTTTTCTGAAATAATTGGTGAAATAAATCTTTAGTGTAAAAGCAGGTGGGTCATTCGAATATCAATATGGTTCAGGGTCATTAAAAAAAAAGTGCGGTTGCGAGCCGCGTGGGATGTGTGGGAGCACTATTTGGGTTCTTTTAAAGTGCGTTACTCGCATGCATGTTTTGTGAAATTTTCAGCCCCACCCCCCATTGTAGGATATTACATTTCATAAAGCAGTTGTTTAATGTCATCTTATTTAGAAATGATGTCATTCAGTTATGAAAACAGTTTGTAAGAAGGCCATTATTATTTTAGAAAGGAGTTGTTTAATGTCATCTTATTTGGAAATGATGTTATTTATTTATTTAGGTATTTTTATATAGCGCTTACCTTAAAGCTCTAAGCGCTTTACAATTATAAAAACATGTAAACTAACTACAATAAAAATGAGACATTAGGATAGTAACTACTATTCAGTTATGAAAACAGTTAGGAAGAAGGCCGTTATTATTTTAGAAAGGATTGCAACAATGCTCACACCCTTGGCAAAATTATTCTGTGACTAAATACGTCATTTTTTTTATGTGTGCTCTTAGCTTATCTAAATTCATATTTCATTTAAGGGATACGCTTAACCACATTACATATTGTTCCTTGTGTCTGAATTTTGATTCAACTGTTAACTTACCTTATGAATGTATGCTATTTGCTTTATATTGCTAAATAATATTATCTTTTCTGGCCAAGCAGAGGGGACATCTATTTGAACTCAGTTCAGTACAGCAATGTCATCACTGATACAGCTTTTTGCTGTTTAGTATTCCATTATCCTGAAGAGATGAGAAAAATTAGTTCCAGGAAGGAATATTGATTGGTGTTAAAACTGCAGAAACGCTGAACAGAAGTTATTTTAACAACAAAATACATATAAATTGTGATCATGTTGTGACTTTGACTTTTGGAAAATGAAATGTAAAAAAATGATTGAAGCCAAACAGATGTAATGCCCATTGGATGCTCAGAAGCATTCTATAGTTGGATACAATGCCCATTGGATGCTCAGAAGCATTCTATATTTGGATACAATTTTAAAATTCCTTTATTTTATTATTAGCATTGAAGACTCATTTTTTCAATATATTTGCATATTTTTGTACAAGTGATCTATTGTCTTTAACAAGCTTTGTGTTTACTATCACTTCACTCCCATGAAATATATTTAATTTATAATTTTATAATTATCAACTGACATTGTTCTATGATCACCACCACTCACTTCCCCTTCATCTAATAACTGATCTATTTTTATTTTCAAAACATTA

**>Lymnaea stagnalis_ p38 alpha homolog_protein**

MADTAQRKPGLVTVELNKTIWEVPSRYKIQNPVGIGAYGQVVSATDNLLCTRVAIKKLARPFQTAIHAKRTYRELRMLRHMNHENVIDLIDVFTPTVTLQDFTDVYLVTPLMGADLNNILKTQRLSDDHVQFLVYQILRGLKYIHSAGILHRDLKPSNIAVNEDCELRILDFGLARLTDEEMTGYVATRWYRAPEIMLNWMHYNQTVDIWSVGCIMAEMLTGRPLFPGSDHIDQLTRILNLVGTPNDELMEEIKSQDAKLFIKSLPVMTRKDFKQVFAGSNPLAIDLLEKMLDLNTKSRLNATQALAHEYLKQYADPADEPVSEKYDMTFEDYDLSINEWKQLVFEEIEKFQAAHQLS*
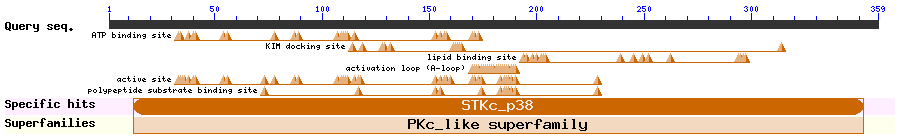


**>Lymnaea stagnalis_JNK1 homolog_mRNA (#MW322712)**

ACAACAAATGTTGACGTCAAACTCAGCAGTCCCTAAATGATGAGTGAGCTAGTGAACGCATTGAGAGTGGAACATCGTCTGACTCATGACAATTGATTTTCTGCATTTTAATTTATCGTTTCTCTATTTCATTTATCTAACATTTAATTTTCTTGGATTAGAGGACAGATATCTTCTCAAGTTAAACCTAATGGGTATTTGAAGACCAACATTTTCTTTGCCAAAATATTTCCTTTTTAAATTGAGAAACATGAGTAAACACACAAACCTCTTTTACACAGTGGAGGTTGGTGATTCCACTTTTACAATACTCAAGCGCTATCAACATCTTAGACCCATTGGATCTGGAGCTCAAGGAATCGTTTGTGCTGCCTGTGACACCCTGACAGACACAAATGTCGCCATCAAGAAACTGAGTCGACCCTTCCAGAATGTGACGCATGCCAAGAGGGCGTACCGTGAATTTGTATTAATGAAACTGGTCAATCATAAAAATATTATTGGATTGCTCAATGCATTCACACCTCAGAAATCCTTGGAGAGCTTTCAAGATGTATATTTAGTGATGGAACTGATGGATGCAAACCTTTGCCAAGTGATACAAATGGATCTTGATCATGAACGTATGTCCTACCTTCTCTACCAGATGTTGTGTGGCATCAAACATTTGCATTCTGCTGGAATAATACACAGAGATCTGAAACCCAGCAACATAGTTGTCAAATCAGATTGTACCCTTAAGATTTTGGACTTTGGATTGGCCAGAACAGCAGGCACAGGTTTCATGATGACACCATATGTTGTAACAAGGTATTACAGAGCTCCAGAAGTTATTTTAGGGATGGGATACAAAGCTAATGTTGATATCTGGTCTGTGGGTTGCATTATGGCAGAACTTATACGAGCAACAGTAATGTTCCCAGGAACTGATCATATTGATCAGTGGAATAAGATAATTGAGCAGCTCGGTACTCCCAGTCAAGAGTTTATGGCCCGCTTGCAAACCACTGTGAGGAATTATGTGGAGAATAGGCCAAAGCACACAGGGTTTAGTTTTGAGAAGTTATTCCCAGATGTGCTCTTCCCTCCTGACAGTGCAGAACACTCAGGCTTGCGTGCCACTGTGGCAAGAGATTTACTATCCAAGATGCTAGTAATAGATCCTGACAAAAGAATTTCAGTAGATGAAGCTCTCAATCACCCCTACATCAATGTATGGTATGATGAACGAGAGGTCAATGGGCCTGCCCCAGGTCCTTATGATCATGCTGTTGATGAACGAGAACACACAGTTGAAGAGTGGAAAACTTTGATATACAATGAAGTGATGGAGTATGAGAATAGGGACAAGAGCAAAGATAAAACAAGTGCTATGCAAAATCACAATGATGGCGGAAATAACATGGAAACAGGGGCAGTGAATGCAGACACTAATGCAACGAGAGCAGCAGCAGCAGCAGCAGCCTCGGCAACAAATGCACTGAACCATCACTAATGATGGCTTTAGCTTGTATATAGATGTAAACCTCCTGGCCCGCCCACCACATATTTCTTTCTGTCATCAGTCAATCCACCTTTCTTTAAATTAACCAGCTCATGTTCCTCATTGTTCCCATTTCTGTTTCTTAGCCCAGGTATTTGGTCGGGACAAGGAGTTTGAGTAAACCAAGCCAAATAATTTCAAAATTAGAAAATAGAAATGATTAAGAATGATTACTTAAGCTGATATGCAATATTTTCATATAAATGTTATATTTTTAATAAAAAAAAGTAATGGGAAATGGCAGATTTTGTACATTTAATTAACACAATTAATTTAATGTAAGTTGTGCTATCTCATAAAGATATAAATTTTAAGTGCTGCTTTTAATTTAAAGCTCATGTGATTTTTCCTTCCTTTTACTCTTATATCTCTGCTTCTAGTCAAATTTGTAGTAATTTAACAAAGAAATATCAATTGGATAGGTTAGCAAGTAATGTTGGCAATATTAATTAATATCTTTGTAGATAATTCAATTGATCAAAAAAGGAATTTCTTTTAATTCCCTGCAGTGAGAAACTTGATTTGAAGGCCAACAACATTTTTAACTTCATTTACGGTCTTTTGTCTAAAATAATTGAAATAGCAAAACGAGGTATGTGGAAGCTTGAATTATAGAACAGGACAAAAAGATACGGACGTGTCTAAAATAGAAAAGTTGTGGTGTTTTACAGAATTTGCTCTTATTTTAAAGTCATCAGCTGAAGTAAATACCCTTAAGAAAAAGAATGCTGCAACATAATATTGCTTAATGTACTTACATTTTTGTGGTTTATTGGTCATCCCAAATCCCATCCTAGTGTAAAGATACATTTCATACTTAAACGGAATGGCTTTGACAAAAGGTATTCAAAGCTTTCAAATATTCTTACTTTTTTTTTTTTTTTAAATAGCATGGGAAAAAAGAT

**>Lymnaea stagnalis_JNK1 homolog_protein**

MSKHTNLFYTVEVGDSTFTILKRYQHLRPIGSGAQGIVCAACDTLTDTNVAIKKLSRPFQNVTHAKRAYREFVLMKLVNHKNIIGLLNAFTPQKSLESFQDVYLVMELMDANLCQVIQMDLDHERMSYLLYQMLCGIKHLHSAGIIHRDLKPSNIVVKSDCTLKILDFGLARTAGTGFMMTPYVVTRYYRAPEVILGMGYKANVDIWSVGCIMAELIRATVMFPGTDHIDQWNKIIEQLGTPSQEFMARLQTTVRNYVENRPKHTGFSFEKLFPDVLFPPDSAEHSGLRATVARDLLSKMLVIDPDKRISVDEALNHPYINVWYDEREVNGPAPGPYDHAVDEREHTVEEWKTLIYNEVMEYENRDKSKDKTSAMQNHNDGGNNMETGAVNADTNATRAAAAAAASATNALNHH*
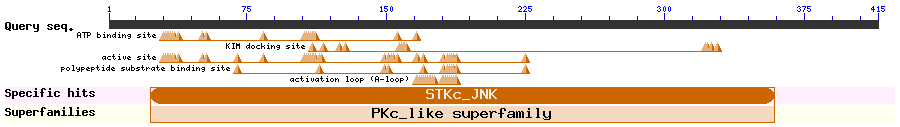


**Sequence comparison between *L. stagnalis* and *Homo sapiens***

#=======================================

#

# Aligned_sequences: 2

# 1: Lymnaea p38

# 2: human p38

# Matrix: EBLOSUM62

# Gap_penalty: 10.0

# Extend_penalty: 0.5

#

# Length: 369

# Identity: 234/369 (63.4%)

# Similarity: 284/369 (77.0%)

# Gaps: 19/369 ( 5.1%)

# Score: 1244.5

#

#

#=======================================

lymnaea 1 MADTAQRKPGLVTVELNKTIWEVPSRYKIQNPVGIGAYGQVVSATDNLLC 50

.:|.:|.....||||||||||.||:..:|||.||||.|.:|.|....

human 1 ---MSQERPTFYRQELNKTIWEVPERYQNLSPVGSGAYGSVCAAFDTKTG 47

lymnaea 51 TRVAIKKLARPFQTAIHAKRTYRELRMLRHMNHENVIDLIDVFTPTVTLQ 100

.|||:|||:||||:.|||||||||||:|:||.|||||.|:|||||..:|:

human 48 LRVAVKKLSRPFQSIIHAKRTYRELRLLKHMKHENVIGLLDVFTPARSLE 97

lymnaea 101 DFTDVYLVTPLMGADLNNILKTQRLSDDHVQFLVYQILRGLKYIHSAGIL 150

:|.||||||.|||||||||:|.|:|:|||||||:|||||||||||||.|:

human 98 EFNDVYLVTHLMGADLNNIVKCQKLTDDHVQFLIYQILRGLKYIHSADII 147

lymnaea 151 HRDLKPSNIAVNEDCELRILDFGLARLTDEEMTGYVATRWYRAPEIMLNW 200

||||||||:||||||||:||||||||.||:||||||||||||||||||||

human 148 HRDLKPSNLAVNEDCELKILDFGLARHTDDEMTGYVATRWYRAPEIMLNW 197

lymnaea 201 MHYNQTVDIWSVGCIMAEMLTGRPLFPGSDHIDQLTRILNLVGTPNDELM 250

||||||||||||||||||:||||.||||:|||:||.:|:.|.|||...|:

human 198 MHYNQTVDIWSVGCIMAELLTGRTLFPGTDHINQLQQIMRLTGTPPAYLI 247

lymnaea 251 EEIKSQDAKLFIKSLPVMTRKDFKQVFAGSNPLAIDLLEKMLDLNTKSRL 300

..:.|.:|:.:|:||..|.:.:|..||.|:||||:|||||||.|::..|:

human 248 NRMPSHEARNYIQSLTQMPKMNFANVFIGANPLAVDLLEKMLVLDSDKRI 297

lymnaea 301 NATQALAHEYLKQYADPADEPVSEKYDMTFEDYDLSINEWKQLVFEEI-- 348

.|.|||||.|..||.||.||||::.||.:||..||.|:|||.|.::|:

human 298 TAAQALAHAYFAQYHDPDDEPVADPYDQSFESRDLLIDEWKSLTYDEVIS 347

lymnaea 349 --------EKFQAAHQLS* 359

|:.::

human 348 FVPPPLDQEEMES------ 360

#---------------------------------------

#=======================================

#

# Aligned_sequences: 2

# 1: Lymnaea JNK1

# 2: human JNK1

# Matrix: EBLOSUM62

# Gap_penalty: 10.0

# Extend_penalty: 0.5

#

# Length: 426

# Identity: 299/426 (70.2%)

# Similarity: 334/426 (78.4%)

# Gaps: 26/426 ( 6.1%)

# Score: 1534.0

#

#

#=======================================

lymnaea 1 --MSKHTNLFYTVEVGDSTFTILKRYQHLRPIGSGAQGIVCAACDTLTDT 48

.||..|.||:||:.|||||:|||||:|:|||||||||||||.|.:.:.

human 1 MSRSKRDNNFYSVEIADSTFTVLKRYQNLKPIGSGAQGIVCAAYDAILER 50

lymnaea 49 NVAIKKLSRPFQNVTHAKRAYREFVLMKLVNHKNIIGLLNAFTPQKSLES 98

|||||||||||||.|||||||||.||||.|||||||||||.||||||||.

human 51 NVAIKKLSRPFQNQTHAKRAYRELVLMKCVNHKNIIGLLNVFTPQKSLEE 100

lymnaea 99 FQDVYLVMELMDANLCQVIQMDLDHERMSYLLYQMLCGIKHLHSAGIIHR 148

|||||:|||||||||||||||:||||||||||||||||||||||||||||

human 101 FQDVYIVMELMDANLCQVIQMELDHERMSYLLYQMLCGIKHLHSAGIIHR 150

lymnaea 149 DLKPSNIVVKSDCTLKILDFGLARTAGTGFMMTPYVVTRYYRAPEVILGM 198

||||||||||||||||||||||||||||.|||||||||||||||||||||

human 151 DLKPSNIVVKSDCTLKILDFGLARTAGTSFMMTPYVVTRYYRAPEVILGM 200

lymnaea 199 GYKANVDIWSVGCIMAELIRATVMFPGTDHIDQWNKIIEQLGTPSQEFMA 248

|||.|||:|||||||.|::...::|||.|:||||||:|||||||..|||.

human 201 GYKENVDLWSVGCIMGEMVCLKILFPGRDYIDQWNKVIEQLGTPCPEFMK 250

lymnaea 249 RLQTTVRNYVENRPKHTGFSFEKLFPDVLFPPDSAEHSGLRATVARDLLS 298

:||.|||.|||||||:.|:||||||||||||.|| ||:.|:|:.||||||

human 251 KLQPTVRTYVENRPKYAGYSFEKLFPDVLFPADS-EHNKLKASQARDLLS 299

lymnaea 299 KMLVIDPDKRISVDEALNHPYINVWYDEREVNGPAPGPYDHAVDEREHTV 348

||||||..|||||||||.|||||||||..|...|.|...|..:||||||:

human 300 KMLVIDASKRISVDEALQHPYINVWYDPSEAEAPPPKIPDKQLDEREHTI 349

lymnaea 349 EEWKTLIYNEVMEYENRDKS---KDKTSAMQNH--NDGGNNMETGAVN-- 391

||||.|||.|||:.|.|.|: :.:.|.:... |...:.:.:.:||

human 350 EEWKELIYKEVMDLEERTKNGVIRGQPSPLGAAVINGSQHPVSSPSVNDM 399

lymnaea 392 --ADTNATRAAAAAAASATNALNHH* 415

..|:.|.|:.

human 400 SSMSTDPTLASD-------------- 411

#---------------------------------------

**CREB – adapted from Sadamoto et al. 2004**

P-box: 85% identity with human

Lymnaea DAKRRREILARRPSYRKILNDLSS - 93

|:::|||||:||||||||||||||

Human DSQKRREILSRRPSYRKILNDLSS - 143

bZIP: 87% identity with human

Lymnaea EEGSRKRELRLLKNREAARECRRKKKEYVKCLENRVAVLENQNKTLIEELKALKELYCQKDA - 264

||.:||||:||:||||||||||||||||||||||||||||||||||||||||||:|||.|..

Human EEAARKREVRLMKNREAARECRRKKKEYVKCLENRVAVLENQNKTLIEELKALKDLYCHKSD - 341

**Acknowledgements**

Bioinformatics infrastructure was supported by ELIXIR Hungary.

**References**

Fodor I, Urbán P, Kemenes G, Koene JM, Pirger Z. Aging and disease-relevant gene products in the neuronal transcriptome of the great pond snail (*Lymnaea stagnalis*): a potential model of aging, age-related memory loss, and neurodegenerative diseases. *Invert Neurosci* 2020 20(3):9. doi: 10.1007/s10158-020-00242-6

Maasz G, Zrinyi Z, Reglodi D, Petrovics D, Rivnyak A, Kiss T, Jungling A, Tamas A, Pirger Z. Pituitary adenylate cyclase-activating polypeptide (PACAP) has a neuroprotective function in dopamine-based neurodegeneration in rat and snail parkinsonian models. *Dis Model Mech* 2017 10(2):127-139. doi: 10.1242/dmm.027185.

Sadamoto H, Sato H, Kobayashi S, Murakami J, Aonuma H, Ando H, Fujito Y, Hamano K, Awaji M, Lukowiak K, Urano A, Ito E. CREB in the pond snail *Lymnaea stagnalis*: cloning, gene expression, and function in identifiable neurons of the central nervous system. *J Neurobiol* 2004 58(4):455-66. doi: 10.1002/neu.10296.

Wick RR, Judd LM, Holt KE. Deepbinner: Demultiplexing barcoded Oxford Nanopore reads with deep convolutional neural networks. *PLoS Comput Biol* 2018 14, e1006583, doi: 10.1371/journal.pcbi.1006583

Lu S, Wang J, Chitsaz F, Derbyshire MK, Geer RC, Gonzales NR, Gwadz M, Hurwitz DI, Marchler GH, Song JS et al. CDD/SPARCLE: the conserved domain database in 2020. *Nucleic Acids Res* 2020 45, pp. D265-D268, doi: 10.1093/nar/gkz991

Marchler-Bauer A, Bo Y, Han L, He J, Lanczycki CJ, Lu S, Chitsaz F, Derbyshire MK, Geer RC, Gonzales NR et al. CDD/SPARCLE: functional classification of proteins via subfamily domain architectures. *Nucleic Acids Res* 2017 45, pp. D200-D203. doi: 10.1093/nar/gkw1129
